# Application of Molecular Methods in the Identification of Ingredients in Chinese Herbal Medicines

**DOI:** 10.3390/molecules23102728

**Published:** 2018-10-22

**Authors:** Ke Han, Miao Wang, Lei Zhang, Chunyu Wang

**Affiliations:** 1 School of Computer and Information Engineering, Harbin University of Commerce, Harbin 150028, China; 2 Life sciences and Environmental Sciences Development Center, Harbin University of Commerce, Harbin 150010, China; w1993817m@163.com (M.W.); 13212921382@163.com (L.Z.); 3 School of Computer Science and Technology, Harbin Institute of Technology, Harbin 150001, China; chunyu@hit.edu.cn

**Keywords:** bioinformatics, identification of Chinese herbal medicines, biochip technology, DNA barcoding technology

## Abstract

There are several kinds of Chinese herbal medicines originating from diverse sources. However, the rapid taxonomic identification of large quantities of Chinese herbal medicines is difficult using traditional methods, and the process of identification itself is prone to error. Therefore, the traditional methods of Chinese herbal medicine identification must meet higher standards of accuracy. With the rapid development of bioinformatics, methods relying on bioinformatics strategies offer advantages with respect to the speed and accuracy of the identification of Chinese herbal medicine ingredients. This article reviews the applicability and limitations of biochip and DNA barcoding technology in the identification of Chinese herbal medicines. Furthermore, the future development of the two technologies of interest is discussed.

## 1. Introduction

The traditional method of determining the authenticity of traditional Chinese medicine is to evaluate the color, shape, or nature of the medicinal materials using either physical/chemical methods or microscopy. These methods are still used, are relatively simple to carry out, and have led to substantial advancements in the screening of Chinese herbal medicines. However, these methods have some shortcomings and disadvantages [1]. It is assumed that there is a certain level of contamination of counterfeit and inferior medicinal ingredients in compound medications made by processing drugs of many ingredients, and it is difficult to identify the contaminating materials following the traditional method of identification. After thousands of years of development, traditional Chinese medicine has grown into a vast system, and its medicinal ingredients are numerous and complex [2]. Moreover, there are many disadvantages to the all too common practices of using the same name to describe many different herbs, or in having multiple names for a single medicinal material. This further increases the difficulty in the identification of Chinese herbal medicines [3,4]. In addition, other conventional Chinese herbal screening methods require the operators to have very rich work experience and expertise, or some subtle changes will lead to errors in the identification results [5]. Due to the error-prone nature of the identification process, it is often difficult to meet actual needs.

Bioinformatics is a discipline that integrates the most advanced knowledge of computer information technology and molecular biology [6,7,8]. This discipline uses the powerful data-processing ability of computers to analyze and compare proteomic, transcriptomic, genomic, and microorganism data, and so on, in order to identify and solve specific problems. At present, this technology has been applied to many fields, including Chinese herbal screening, as described in this review. It has also been applied to other medical fields, such as studying the effect of a drug on the prevention and treatment of diseases, and studying special drugs for cancer cell proliferation and death [9]. Therefore, it is of great significance and value for Chinese medicine modernization to further study the identification of Chinese herbal medicines by bioinformatics.

At present, bioinformatics assists in the identification and assessment of Chinese herbal medicines through the use of DNA barcoding technology and biochip technology. DNA barcoding seeks to identify biological material through sequencing a selected genetic marker, and then comparing that DNA sequence to sequences from the same genetic marker in other species [10,11]. Nowadays, this technology has been applied widely, and can be regarded as a mature tool for biological exploration and research. Not only can it be used to identify organisms that are difficult to distinguish by other methods, it can also be used to discover new organisms that have not been discovered by the biological research community to date [12]. If a specimen of a suspected new organism is collected, this technique can be used to extract its corresponding DNA barcode, and then compare and analyze it with DNA barcodes (nucleotide sequences) that are available in the public nucleotide sequence database, so that the relevant identity information of this organism can be determined step-by-step. On the other hand, biochip technology is a microarray that is composed of some biological components (nucleic acids, proteins, cells, etc.) wrapped on solid supports such as nylon film or a silicon wafer [13]. Through the use of a chip, this technology takes advantage of automation, speed, and big data. This review discusses the promotion and use of bioinformatics in the field of Chinese herbal medicine identification in recent years, and compares the advantages and disadvantages of various methods. With the broad applicability of these techniques, we hope that the identification of Chinese herbal medicines can be further developed.

## 2. Biochip Technology

Since the 1990s, due to continuous progress and development in science and technology, especially the rapid development of computer and network technology, a series of related disciplines emerged [14,15,16,17,18]. This also led to the development of biological chip technology for microanalysis research [19]. Biochip technology is a comprehensive technology discipline that combines chemical and physical technology. Biochip is the crystallization of DNA hybridization probe technology combined with semiconductor industry technology. The technique is to hybridize a large number of probe molecules with fluorescent-labeled DNA or other sample molecules (e.g., proteins, factors, or small molecules) after immobilizing them on the support. The number and sequence information of the sample molecules can be obtained by detecting the hybridization signal intensity of each probe molecule [20]. Fluorescence-labeled target molecules play a role in a variety of microbodies on the chip. A spectrophotometer is used for analysis of spectral/absorption characteristics, and the results will vary according to the intensity of the material. Next, a special instrument is used to collect and convert the data to be processed by a computer. In this way, the required biological information will be obtained [21,22]. Nowadays, biochips can be divided into tissue chips [23], protein chips, and gene chips. The latter two are widely used in Chinese herbal medicine identification.

### 2.1. Gene Chip

**Figure 1:**
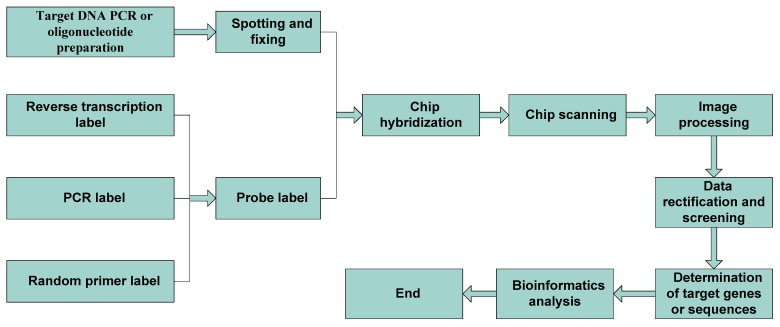
Steps of gene chip technology.

Gene chip technology is fast, highly efficient, automated, parallel, and economical. It has become an important technical method in the field of screening and the evaluation of inferior and counterfeit drugs of Chinese herbal medicine. The process involves obtaining the standard atlas of positive drugs and the atlas of prepared identification (query) drugs, and then analyzing and comparing the differences between the two. To obtain the two maps, one must first extract DNA from the corresponding samples, and then let them hybridize with DNA chips [24,25,26,27]. The detection steps of gene chip technology are shown in Figure 1.

Zhang et al. [28] used gene chip technology to study the *Dendrobium nobile* (*Orchidaceae, Dendrobium Sw.*) species mixed in compound medication, and five species of *Dendrobium nobile* were successfully detected and loaded into China Pharmacopoeia (https://www.sinicave.com/pd_pharmacopoeia.cfm). It has been shown that this technique can be used in the field of Chinese herbal medicine taxonomy. Hao et al. [29] was the first to use AFLP (amplified fragment length polymorphism) technology in the study of the characteristics of Chinese herbal medicines, after which they drew the atlas of genetic diversity and AFLP fingerprints on the target taxa. This method can be used to easily and effectively differentiate between the characteristics of Chinese herbal medicines. The AFLP marker is then turned into a SCAR (sequence characterized amplified regions) marker, and the mounting glue can be recycled, or molecular cloning by PCR can be performed. The obtained PCR can be used to detect the characteristics of the target medicinal ingredient. Among expectorant and antitussive drugs, *Fritillaria cirrhosa* (*Liliaceae*, *Fritillaria*) has a positive effect, and is favored by many [30]. However, the high price of the highly sought-after drug encouraged sales of counterfeit or low-quality replicas at lower prices. In view of this, some scholars [31] began to sequence and study the 5S rRNA gene of *Fritillaria hupehensis*, *F. anhuiensis*, *F. thunbergii*, and *Fritillaria cirrhosa* by gene chip technology, and found that *Fritillaria cirrhosa* has a unique sequence of 5′-CTTTTGTCGATCA-3′, which is absent in other *Fritillaria* species. This sequence was used to make the gene chip. This technique is used to detect and extract the gene sequences of some tested products, and then compare them with the gene sequence of the positive *Fritillaria cirrhosa* control, to determine whether the sample that is being tested contains the expected product.

In another study, Chen et al. [32] isolated the genomic DNA of *Coptis chinensis* (*Ranunculus*, *Coptis Salisb.*) as a template, and then analyzed the many thermal cycle parameters in the ISSR (inter-simple sequence repeat) reaction system using two factors and single factors, as well as the effect and influence of some major components of amplification to identify the optimal conditions. Finally, the reaction system and amplification procedure that are suitable for the ISSR analysis of *Coptis chinensis* were established under optimum conditions. The establishment of this optimization system provides a standardized procedure for the identification and genetic diversity analysis of *Coptis chinensis* by the ISSR marker technique in the future.

The greatest advantage of a gene chip is high throughput. The process of marking and hybridizing probes for many genes can be completed in a single experiment; the degree of automation is high, and the data are objective and reliable. However, its greatest shortcoming is that the gene chip cannot be used to find new things; it can only be used to find things that have been found before (and have been printed on the chip).

### 2.2. Protein Chip

Protein chip technology is mainly used for protein analysis and exploration. The methodology involves the use of advanced microelectronics technology to analyze the surface of some carriers in order to establish a system that is suitable for microbiological research [33,34,35]. First, some known proteins are fixed to the carrier in order; then, the molecules can specifically interact with known molecules based on molecular properties, after which the molecules are ready for purification and subsequent treatment. Finally, the protein components can be more quickly and accurately screened [36,37].

Li et al. [38] used an NP10 chip and a protein chip combined with SELDI-TOF MS (surface-enhanced laser desorption/inionation-time of flight-mass spectra) to analyze the peptide composition and protein of tortoise shell glue. The peptide component/protein quality fingerprint of tortoise shell glue was obtained. This technology can be used for the digital analysis of tortoise shell glue. Wang et al. [39] obtained the protein/peptide from the dried and processed *Pheretima asiatica*, and analyzed the protein/peptide information with the surface-enhanced laser desorption/ionization time-of-flight mass spectrometry technique. Twenty-nine peaks of protein/peptide molecular weight were obtained. Among them, crude and dried *Pheretima asiatica* had 17 peaks of meaningful molecular weight, and processed *Pheretima asiatica* had 12 peaks. There was an amino acid residue among multiple adjacent protein peaks, and multi-groups of molecular weight of crude and processed *Pheretima asiatica* were extremely similar, indicating that these peptides may represent the same peptides. Molecular weight fingerprinting is obtained from the crude and processed *Pheretima asiatica* protein/peptide by laser desorption/ionization time-of-flight mass spectrometry; it can serve as a digitalized quality and control standard of *Pheretima asiatica*, and provide references for the further isolation, purification, and verification of the proteins/peptides associated with *Pheretima asiatica* function.

Protein chip technology has the following advantages: it is low-cost because it requires only a few samples and reagents in the process, it is more accurate and sensitive than conventional ELISA (enzyme linked immune sorbent assay), it is easy to operate due to automation, it is highly accurate, a large number of proteins can be quickly analyzed, and it has few sample requirements. The sample to be tested can be detected and analyzed by a protein chip only by simple processing. However, this technology has some shortcomings compared to gene chip technology: the purification of protein is more difficult, there is a lack of mature protein technology, the technology is inseparable from the function and re-modification of other proteins, and proteins are more variable among themselves. Therefore, protein chips are used less frequently than gene chips.

## 3. DNA Barcoding

**Figure 2:**
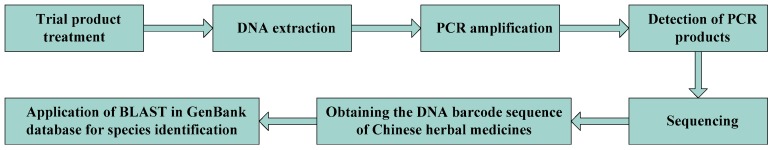
Molecular identification process of DNA barcoding in Chinese medicinal materials.

Ever since Canadian scientist Paul Herbert [40] proposed DNA barcoding technology back in 2003, this diagnostic technology has been widely used in the field of species identification. DNA barcoding uses a short, unique DNA sequence to identify a species. Since traditional Chinese medicines are derived from a variety of animals, plants, and minerals, this technology can be used for the accurate identification and evaluation of medicinal ingredients. This technique is widely used in the field of biological and medicinal material identification because it is not influenced by the morphology of the sample or the surrounding environment [41]. The application of this technology has broadened widely, and has reached multiple fields and industries, such as ecology, development, and evolution, Chinese herbal medicine identification, and genetic identification [42]. A non-trivial proportion of the public fungal DNA sequences are compromised in terms of quality and reliability, contributing noise and bias to sequence-borne inferences. R. Henrik Nilsson et al. [43] discussed various aspects and pitfalls of sequence quality assessment. Based on their observations, they provided a set of guidelines to assist in the manual quality management of newly generated, near-full-length (Sanger-derived) fungal internal transcribed spacer (ITS) sequences, and to some extent also sequences of shorter read lengths, other genes or markers, and groups of organisms. A flow chart describing the process of the DNA barcoding molecular identification of Chinese herbal medicines is shown in Figure 2.

According to the selection of different DNA sequences, DNA barcoding can be roughly divided into four categories: (1) mitochondrial DNA barcode, (2) ribosomal DNA barcode, (3) chloroplast DNA barcode, and (4) DNA barcode combination identification. A suitable DNA barcode should meet the following conditions: high throughput so that it can be routinely sequenced in plant species, suitable for producing a high-sequence mass coverage of bidirectional sequences with minimal unsequenced bases, high resolution so that most species can be distinguished, and DNA fragments should be short enough so that degraded DNA can be amplified.

### 3.1. Mitochondrial DNA Barcode

Mitochondrial DNA barcode technology was the first technique to be used in the classification and identification of animal mitochondria. Mitochondrial COI (cytochrome oxidase) gene barcode technology has been an important method in identifying animal Chinese herbal medicine [44]. Hebert [40] eventually selected COI sequences, because COI sequences ensured sufficient variation and were easy to be amplified by universal primers, and there were few deletions and insertions in its own DNA sequence, so it was suitable for the analysis of closely related taxa.

Shi Linchun et al. [45], in order to distinguish *periostracum serpentis*(the skin that the snake shed, PS) from its adulterants, PCR amplified and sequenced COI sequences of 68 samples from 13 species. Furthermore, the DNA barcoding gap and phylogenetic cluster analysis were carried out. The results showed that three specimens of *periostracum serpentis Elaphe taeniura* (Cope), *E. carinata* (Guenther), and *Zaocys humnades* (Cantor)—had DNA barcode gaps, and they were separated into independent branches on the neighbor-joining (NJ) system clustering tree. As a DNA barcode, COI can not only identify three basic genera and species of Chinese herbal snakeskin, it can also distinguish between snakeskin and their easily confused products. This shows that DNA barcoding can be used for the identification of the snake shedding that is found in Chinese medicinal products. Zhang Hongyin et al. used the same method to identify the pseudo products of *centipede* [46], *deer medicine* [47], and *Gekko gecko* [48]. The results showed that the COI gene can be used as an effective marker for identifying at least metazoan Chinese medicinal ingredients.

The COI gene sequence exists in the vast majority of animal cells. There are only one set of genomic chromosomes in a cell, whereas there are hundreds of mitochondria per cell; that is why mitochondrial DNA is more easily recovered. The mitochondrial DNA mutation rate is 10 times greater than that of genomic DNA. According to this characteristic, it is easier for us to accurately differentiate species. However, one disadvantage of this technology is that it requires a lot of tedious work in the identification of species, and its identification results are not produced directly or quickly.

### 3.2. Ribosomal DNA Barcode

The nuclear ribosomal ITS (internal transcribed spacer) region contains the ITS1 intergenic region, the ITS2 intergenic region, and the 5.8S gene (ITS1-5.8S-ITS2) ranging in size from 400 bp to over 1000 bp. ITS has a high capacity for species identification and technical scalability. Ribosomal DNA is a polygene family, and ITS exists in highly repetitive ribosomal DNA [49,50]. Due to its fast evolution speed and short length, this genetic marker is widely used in the field of angiosperm branching analysis in plant systems and fungal metabarcoding [51].

Li et al. [52] used ITS primers to amplify the ITS sequences of *Hedyotis diffusa Willd* (*Rubiaceae, Cerastium*) and *Corymbose Hedyotis Herb* (*Oldenlandia corymbosa* L.), and found that there are obvious differences in the ITS sequences between the two plants. They then used the established phylogenetic tree to analyze the common *Herba Hedyotis* that is available on the market, and found that only the medicinal herbs purchased from Guangzhou were genuine; those purchased from Boston and Hong Kong were actually *Corymbose Hedyotis Herb*. Moreover, there was confusion regarding the samples that were used in previous studies of medicinal materials, indicating that it is difficult to distinguish between the two types of medicinal materials based on their shape and characteristics. Thus, the identification of Chinese medicinal materials assisted by bioinformatics is more accurate than the traditional identification method. Based on ITS2 barcode technology, Yu Junlin et al. [53] explored 17 samples of *Bupleurum longiradiatum Turcz* and 31 samples of *Bupleurum* species. They examined the intraspecific variation of two species by analyzing and studying two categories, and identified an accurate distinction between two major species. In order to ensure the accuracy of the study, the samples used in the study have been validated using BLAST (Basic Local Alignment Search Tool) following the DNA barcoding identification system for Chinese medicinal materials (http://www.tcmbarcode.cn). The ITS2 barcode sequence can accurately identify *Bupleurum* and *B. longiradiatum Turcz*. The minimum kimura 2-parameter(K2P) distance between *Bupleurum* and *B. longiradiatum Turcz* is far greater than the maximum K2P distance within the species of *Bupleurum*, and the NJ tree shows that the *B. longiradiatum Turcz* constitutes a single branch, which can be distinguished from *Bupleurum*. Therefore, *Bupleurum* and *B. longiradiatum Turcz* can be distinguished consistently and accurately using the ITS2 barcode. Shi Yuhu et al. [54] used the ITS2 sequence as a barcode to identify the herbal tea ingredient *Plumeriarubra* and its adulterants. Genomic DNAs from 48 samples were extracted; the ITS2 sequences were amplified and sequenced bidirectionally; and then they were assembled and obtained using CodonCode Aligner (https://www.codoncode.com/). The sequences were aligned using ClustalW, the genetic distances were computed by the K2P model, and the NJ phylogenetic tree was constructed using MEGA5.0. The results showed that the length of the ITS2 sequence of *P. rubra* were 244 bp. The intraspecific genetic distances (0–0.0166) were much smaller than interspecific ones between *P. rubra* and its adulterants (0.3208–0.6504). The NJ tree indicated that *P. rubra* and its adulterants could be distinguished clearly. Therefore, using the ITS2 barcode can accurately and effectively distinguish the herbal tea ingredient *P. rubra* from its adulterants, which provides a new molecular method to identify *P. rubra* and ensure its safety in use.

ITS sequences are usually used to distinguish some species that are closely related to each other. It may be difficult to identify the different geographical distribution and host types of some fungi. Not all 18 S and 28 rRNA databases have been established, and many of the data are still lacking, which results in the inability to rapidly identify the required ITS fragments, thus inevitably affecting the application of ITS methods.

### 3.3. Chloroplast DNA Barcode

psbA-trnH intervals in common flowering plants are between 340–660 bp. This sequence interval was compared with nine other genetic markers (matK, rbcL, and ITS are included) with a discrimination efficiency of 83% and an amplification efficiency of 100% [55,56,57,58]. Zhang Yaqin et al. [59] used psbA-trnH sequence technology to study and explore *Pyrrosia* in order to distinguish the appearance and form of counterfeit herbs that are similar to those of *Pyrrosia*. They collected partial sequences of psbA-trnH in the chloroplast genes of *Pyrrosias* and some counterfeit products. Next, the group used two conventional methods based on the partial sequence of the collected psbA-trnH: the minimum distance method and the similarity search method. It was found that the former method could not distinguish between two *Pyrrosia* species; however, the use of the psbA-trnH sequence clearly distinguishes some other counterfeit products mixed with other kinds of *Pyrrosias*. This study shows that the psbA-trnH sequence can effectively distinguish and identify genuine and counterfeit products of pteridophytes.

As one of the fastest evolving regions in plant chloroplasts, the matK gene is about 1500 bp [60]. Hilu and Lahaye [61] also pointed out that it can be implemented in a single fragment when choosing a barcode of a plant. The fragment is then added according to the complexity of the group. Genievskaya Y et al. [62] identified species defined by morphological traits using sequences of the nuclear ribosomal DNA ITS1-5.8S-ITS2 region and matK. The polymorphic sequence positions in Kazakh populations and GenBank (Benson et al. 2017) references were acquired by comparison with GenBank sequences, which identified a difference between local populations of sand rice (*Agriophyllum squarrosum*). ITS and matK sequence analysis revealed a segregation of *Agriophyllum squarrosum* (L.) *Moq* from *A. minus* into separate branches in maximum-likelihood dendrograms. ITS analysis can be used to characterize the populations of *A. squarrosum* growing far away from each other. The data obtained in this study laid the foundation for the further study of *A. squarrosum* populations, and summed up the advantages and disadvantages of this technology. Wang Xiaoming et al. [63] used the matK sequence method in their analysis and exploration of *Herba Abri* (*Leguminous*, *Abrus* L.), and summarized the advantages and disadvantages of this technology in the applications of the DNA barcode in this plant. The modified CTAB (Cetyltriethyl Ammnonium Bromide) method was used to extract the total DNA of nine kinds of *Herba Abri* from different regions, and the matK sequence was amplified using the universal primers of leguminous plants. After that, both K2P genetic distance calculation and the creation of the NJ tree showed that the ITS2 sequences can be used as the DNA barcode sequence of the *Fabaceae* plants. The results showed that the total length of the matK sequence was 889–895 bp; the genetic distance between different plants was far greater than the genetic distance within the populations of the same plant, and even the smallest interspecific genetic distance still exceeded the maximum intraspecific genetic distance. Therefore, matK sequence technology can serve as a DNA barcode for leguminous plants.

rbcL is a fragment of the coding region of a chloroplast gene. The rbcL fragment has a low species resolution, but it is of relatively high species resolution in angiosperms [64,65,66]. Chen Jianxiong et al. [67] collected nine samples of Chinese lobelia of different origins and seven samples of *Mazus japonicus*, and extracted the total DNA from all of the samples collected. The rbcL fragment in the chloroplast DNA of the sample was sequenced, and Clustal X 2.1 software (University College Dublin, Dublin, Ireland) was used for multiple sequence alignments. The NJ clustering feature of MEGA 5.0 software (Center of Evolutionary Functional Genomics Biodesign Institute Arizona State University, Phoenix, AZ, USA) was then used for cluster analysis. They designed specific primers identified by SNP micropoints for two groups of samples, established a specific PCR identification method, and used SYBR Green I (Molecular Probes) dye to establish a rapid detection method for two kinds of Chinese herbs. They successfully identified the Chinese lobelia and *Mazus japonicus*. The results showed that rbcL has strong molecular identification ability and rbcL is easy to amplify and compare. So, rbcL was selected as the DNA barcode for species identification. Huang Qionglin et al. [68] identified *Nervilia fordii (Hance) Schltr*. and its adulterants byrbcLsequencing. They used a commercial kit to extract genomic DNA from fresh leaves; PCR amplification and sequencing were conducted with a pair of universal primers. DNAMAN (https://www.lynnon.com/); Clustal X (University College Dublin, Dublin, Ireland) and MEGA 4.0 software (Center of Evolutionary Functional Genomics Biodesign Institute Arizona State University, Phoenix, AZ, USA) were used for sequence alignment, genetic distance analysis, and clustering analysis. They acquired 502 bp sequences of the rbcL gene from *N. fordii* and its adulterants. Three types of *N. fordii* showed completely consistent sequence data, and differences in five sites were shown between *N. fordii* and *N. plicata*. The interspecific variations were larger than the intraspecific ones. In the cluster dendrogram, all of the species were monophyletic and distinguished from the others. The results showed that the rbcL gene can be used as a DNA barcode to identify *N. fordii* and its counterfeit.

### 3.4. DNA Barcode Combination Identification

Studies have found that it is difficult to identify some species accurately if only one genetic marker is used in the identification of Chinese herbal medicines. Therefore, it is not possible to rely on a single genetic marker to identify all of the species, especially for those species with complex genetic backgrounds [69,70,71,72]. Therefore, a combination of DNA barcodes can be used to accurately identify species.

The CBOL Plant Working Group [73] found that rbcL and matK were suitable barcodes for plants, because these two barcodes were used to successfully identify 550 species from 907 samples, with a success rate of roughly 72%. Moreover, the barcode can become a basic criterion for the molecular identification of species, and it can also provide references for discovering hidden species. Yong et al. [74] tested the universality of rbcL, matK, trnH-psbA, and ITS as DNA barcodes for tree species, and then examined the accuracy of the phylogenetic inference and species identification in three tropical cloud forests (Table 1). Their results suggested that rbcL and trnH-psbA should be adopted as the standard DNA barcode for tree species in tropical cloud forests. The success rates of identifying four fragments were all higher than 41.00%, demonstrating that these fragments are candidates for use in species identification. They used random fragment combinations of rbcL, matK, and trnH-psbA to infer phylogenetic relationships, and established the optimal evolutionary tree with high supporting values in tropical cloud forests.

Priyanka et al. [75] studied the steno-endemic species of the genus *Decalepis* (*Decalepisar ayalpathra Venter*, which is locally known as Amirthapala, is a steno-endemic species in the eastern and western ghats of peninsular India), and found the corresponding DNA barcode, which can be used to monitor and stop the illegal trade of these endangered species. They used rbcL, matK, psbA-trnH, ITS, and ITS2 as DNA barcode candidates. The average intraspecific variation was 0–0.27%, which was less than the distance to the nearest neighbor (0.4–11.67%) with ITS and matK. Finally, they combined rbcL, matK, andITS to produce 100% species resolution using the PAUP (http://paup.phylosolutions.com/, Phylogenetic Analysis Using PAUP) and BOLD (http://v3.boldsystems.org/, Bold systems) methods with the least number of marker combinations to support a character-based approach. They found that the most advantageous barcode datasets were achieved by combining rbcL, matK, ITS, mat, ITS, and ITS2, with a consistency index (CI) of 85% and 90%, respectively. They included 2106 characters in the former dataset for parsimony analysis, among which 103 were parsimony informative, and 18 variable characters were parsimony-uninformative. The 1836 total characters of the latter dataset contributed 146 informative characters. Therefore, the rbcL, matK, and ITS combination is considered to be the best choice for species resolution in the genus *Decalepis*. DNA barcoding has greatly improved species identification and resolution.

Compared with use of single DNA barcodes for identifying species, combining DNA barcodes can greatly improve species resolution. However, researchers need to find the right combination of DNA barcodes. The use of DNA barcode combinations for species identification is more complicated and costlier than use of a single barcode. Therefore, different identification methods should be selected for different species, so as to achieve quick and convenient identification results.

### 3.5. The Limitations of DNA Barcoding

Although DNA barcoding has many advantages in the identification of Chinese herbal medicine, it still has some limitations. In fungi, the ITS region has been sequenced for something similar to 1% of the estimated number of extant species. This makes it tricky to compare newly generated sequences to the entries in GenBank. First, traditional Chinese herbal medicines are usually made of dead animals and plants. Due to collection, processing, storage time, and storage conditions, the DNA macromolecules in the test sample may have been destroyed and degraded prior to analysis. Second, it is difficult to obtain the template that is needed to identify DNA molecules when only small fragments are retained in the sample. Third, although DNA barcoding technology can effectively identify and evaluate plant and animal material in medicinal ingredients, the technology cannot identify the mineral components. Finally, DNA barcoding technology requires technical expertise and is costly.

In order to solve these problems, we should promote basic research, enrich the genome sequencing data, and find small fragments of molecular markers. When dealing with medicinal materials, we should strictly follow standard methods and conditions to avoid damaging the DNA. Results can be validated by using multiple fragments or molecular markers to obtain more accurate DNA fragments, and we should simply test the plant or animal tissues or extracts upfront, before they are used to make medicines. Previous researchers used ClustalW to multiple sequence alignment, but this method is a tool that is long-since obsolete. The user should go for a recent (and readily updated) one, rather than relying on old programs. We recommend any of MAFFT (Katoh et al. 2010), Muscle (Edgar et al. 2004), and PRANK (Löytynoja et al. 2005) for large or otherwise non-trivial sequence datasets [76]. For phylogenetic analysis, NJ is a long-since obsolete tool; Bayesian inference in MrBayes is a whole lot more powerful. In future research, researchers can use these better tools to improve identification.

## 4. Future Perspectives

Chinese medicine reveals a rich history of our people’s long-term struggle against diseases, and has made great contributions to the prosperity and rebirth of the Chinese nation. Starting from Shennong’s Herbal Classic (https://en.wikipedia.org/wiki/Shennong_Ben_Cao_Jing), Chinese medicine plays a very important role in our historical society. However, after a period of instability, the development of traditional Chinese medicine is regrettably at a standstill. With improvements in people’s standards of living and access to technology, medical practitioners and researchers in China and abroad have taken a new interest in Chinese herbal medicine. However, this increased interest in traditional Chinese medicine has led to the emergence of counterfeit and low-quality Chinese herbal medicine in the market, which has greatly reduced the efficacy of Chinese herbal medicine. Therefore, it is essential that we develop a fast and accurate method to identify counterfeit and low-quality Chinese herbal medicines.

Chinese herbal medicines can be identified based on three aspects: appearance, chemical composition, and molecular characteristics. Many medicinal herbs are similar in appearance and are not easy to differentiate, which increases the risk of false identification. Since many Chinese herbal medicines are made up of many kinds of materials that are subsequently ground into a powder, it is very difficult to identify Chinese medicinal ingredients according to chemical constituents. Bioinformatics has brought new opportunities for the identification of traditional Chinese herbal medicine ingredients using high throughput and big data. At present, there are mainly two methods of bioinformatics identification of Chinese herbal medicine: biochip technology and DNA barcoding. The use of DNA barcoding is more extensive, because this method has different identification methods for different kinds of Chinese herbal medicines, making it a more targeted approach. Moreover, the combination identification method of DNA barcoding allows for more species to be identified accurately. The first step toward the DNA barcoding of Chinese herbal medicine is to extract different DNA fragments from different species and select the most suitable DNA fragment, followed by PCR amplification to detect PCR products. Finally, the target bands should be bidirectionally sequenced by DNA sequencing. This method is not without its limitations, and cannot be used to identify all of the species. Therefore, future studies should combine traditional Chinese medicine identification methods, biochip technology, DNA barcoding technology, and high-throughput sequencing-driven metabarcoding to identify Chinese medicinal ingredients and obtain more accurate and rapid identification results. We believe that through the continuous efforts of researchers, the bioinformatics-assisted identification of Chinese herbal medicines will make substantial advancements. It will one day be possible to quickly identify counterfeit and inferior medicinal ingredients from a large number of mixed Chinese medicinal materials, thus standardizing the market of Chinese herbal medicines.

## Figures and Tables

**Table 1:** Results of PCR amplification success rate and DNA sequencing rate for rbcL, matK, trnH-psbA, and internal transcribed spacer (ITS), respectively.

	RbcL	MatK	TrnH-psbA	ITS
Success rates of PCR amplification	75.26% ± 3.65%	57.24% ± 4.42%	79.28% ± 7.08%	50.31% ± 6.64%
Rates of DNA sequencing	63.84% ± 4.32%	50.82% ± 4.36%	72.87% ± 11.37%	45.15% ± 8.91%
